# Editorial: Industrial application of extreme microbes: harnessing the power of nature's extremophiles

**DOI:** 10.3389/fmicb.2025.1697504

**Published:** 2025-09-22

**Authors:** Sahib Zada, Muhammad Rafiq, Wasim Sajjad

**Affiliations:** ^1^Guangzhou Institute of Energy Conversion, Chinese Academy of Sciences, Guangzhou, China; ^2^Department of Microbiology, Balochistan University of Information Technology, Engineering and Management Sciences, Quetta, Pakistan; ^3^Key Laboratory of Cryospheric Science and Frozen Soil Engineering, Northwest Institute of Eco-Environment and Resources, Chinese Academy of Sciences, Lanzhou, China

**Keywords:** extremophil, industrial application, extremozyme, bacteria, microbes

**Figure 1:**
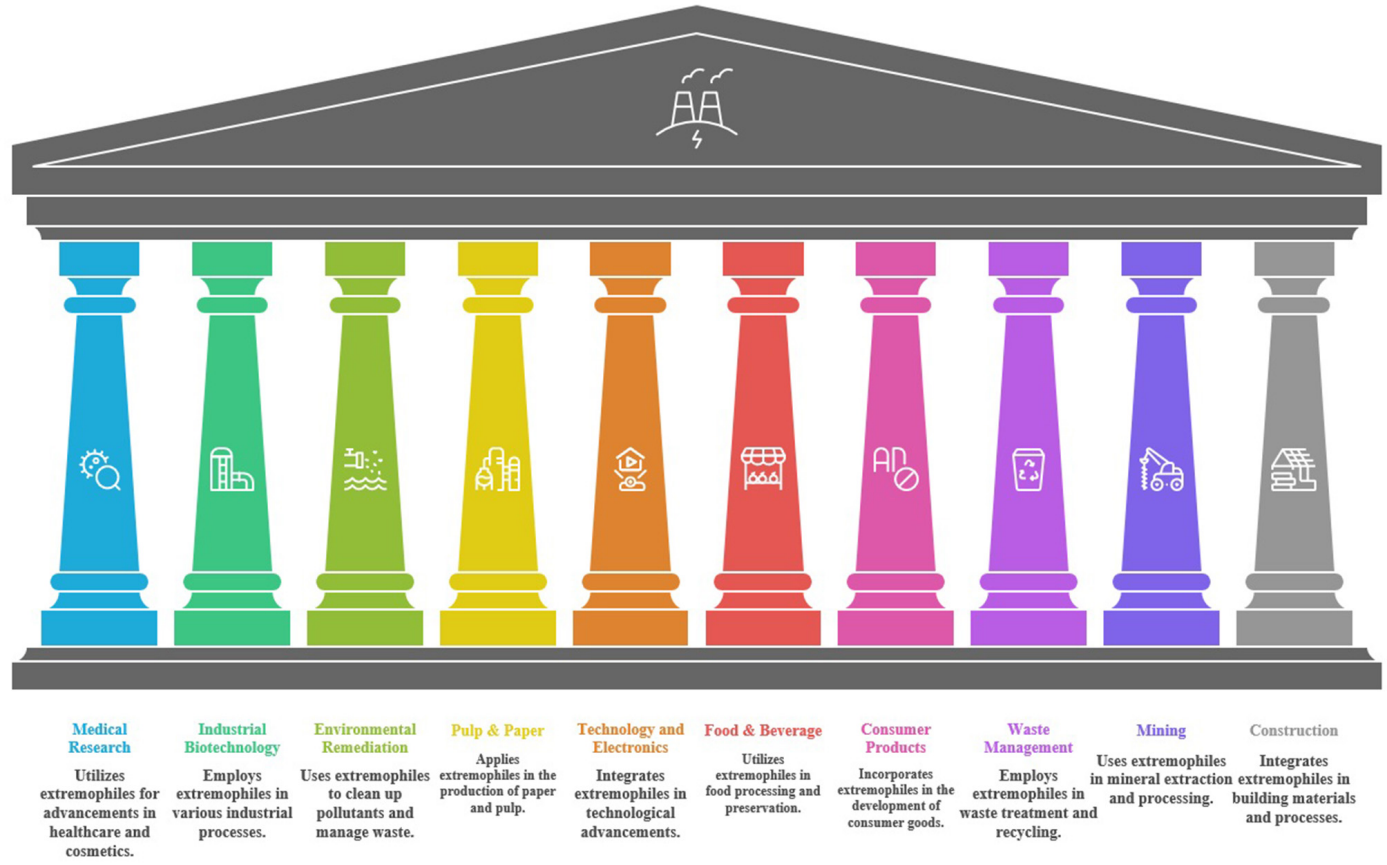
Applications of extremophiles in various industries.

As industrial biotechnology advances toward robust and sustainable solutions, the Research Topic *Industrial application of extreme microbes: harnessing the power of nature's extremophiles* presents a timely compilation of studies demonstrating the vast potential of extremophiles, organisms thriving under extreme conditions, for industrial innovation. Contributions from research groups across Pakistan (1), China (4), and USA (2) highlight a global effort to transform the unique biological adaptations of these microbes into practical applications spanning renewable energy, environmental remediation, food processing, and biomedical sciences (Figure 1).

Biofilms, complex microbial consortia encased in self-produced extracellular matrices, represent a key survival strategy in extreme environments. Bhat and Roach provide a comprehensive review of extremophilic biofilms, underscoring their structural resilience, cooperative interactions, and nutrient acquisition mechanisms. Beyond ecological significance, these biofilms harbor novel bioactive compounds with therapeutic and antimicrobial potential, positioning them as valuable biotechnological resources.

Hot spring ecosystems serve as reservoirs of diverse and functionally versatile microorganisms. Chen et al. characterized bacterial diversity across 11 hot springs in Guizhou Province, China, identifying dominant phyla such as *Pseudomonadota, Bacillota, Nitrospirota, Bacteroidota*, and *Actinomycetota*. Functional predictions revealed enriched pathways in amino acid and carbohydrate metabolism, secondary metabolite biosynthesis, and stress adaptation, offering insights into microbial evolution and providing a genetic blueprint for biocatalyst discovery and biogeochemical modeling.

Enzyme engineering represents another promising avenue. Yan et al. cloned and expressed a uricase gene (*truox*) from *Thermoactinospora rubra* YIM 77501T. The resulting enzyme, TrUox, exhibited high catalytic efficiency at neutral pH and remarkable thermostability, maintaining activity after 4 days at 50 °C. In hyperuricemic models, TrUox effectively reduced serum uric acid levels, while molecular dynamics simulations confirmed its structural rigidity and global stability compared with Rasburicase. These findings establish TrUox as a robust candidate for industrial-scale biocatalysis and therapeutic applications.

Sistu and Holden explored biohydrogen production by the hyperthermophilic archaeon *Thermococcus paralvinellae* using brewery wastewater. Formate supplementation enhanced hydrogen yields, particularly during mid-logarithmic growth, without altering hydrogenase or formate hydrogenlyase activities. This study demonstrates the feasibility of coupling extremophile metabolism with industrial waste valorization, advancing biohydrogen as a renewable energy source.

For environmental remediation, Liang et al. investigated cadmium-resistant strains of *Bacillus cereus* capable of sequestering cadmium and exhibiting resistance to multiple heavy metals. These strains displayed additional adaptive traits, salt tolerance, siderophore production, and metabolic versatility, underscoring their immediate applicability for bioremediation of contaminated soils and waters. These strains do more than just survive; they actively sequester the heavy metal, showcasing a clear and immediate potential for microbially assisted cleanup of contaminated soils and water.

In the domain of food biotechnology, Zhao et al. reviewed mixed-strain fermentation processes, particularly involving extremophiles. Mixed fermentations enhance sensory complexity, stabilize product quality, and expand flavor profiles by leveraging synergistic microbial interactions. Extremophilic enzymes (extremozymes) offer additional advantages such as salt tolerance and thermostability, highlighting their role in overcoming the limitations of conventional fermentation. This review highlights the role of extremophilic microorganisms in overcoming the limitations of traditional fermentation processes.

Finally, Sikandar et al. examined the micellization behavior of rhamnolipids produced by a thermophilic *Pseudomonas aeruginosa* strain isolated from oil field environments. Critical micelle concentration (CMC) values were shown to depend on temperature and salinity, with corresponding changes in thermodynamic parameters (ΔG°, ΔH°, ΔS°). Antimicrobial assays revealed enhanced pathogen inhibition under varying salt conditions, providing mechanistic insights into how extreme environments modulate supramolecular structures and bioactivity of biosurfactants. The research deciphers how extreme temperatures and salinity affect the supramolecular arrangements and micellization of these compounds.

Collectively, these contributions emphasize that extremophiles are not merely biological curiosities but valuable reservoirs of enzymes, pathways, and metabolites with broad industrial relevance. As genetic, computational, and bioprospecting tools advance, the translation of extremophilic adaptations into practical applications is accelerating, offering sustainable strategies for energy, health, environment, and industry.

